# Emergence of multidrug resistant *Enterobacter* spp. in Nigeria: a genomic study

**DOI:** 10.1007/s11274-026-05324-y

**Published:** 2026-10-07

**Authors:** Ayodele Timilehin Adesoji, Vittoria Mattioni Marchetti, Claudia Cortimiglia, Francesca Piscopiello, Ilaria Petrizzi, Aurora Piazza, Emmanuel Dayo Alabi, Pier Sandro Cocconcelli, Roberta Migliavacca

**Affiliations:** 1 Department of Microbiology, Federal University Dutsin-Ma, Dutsin-Ma, Katsina State Nigeria; 2 Department of Microbiology, Babcock University, Ilishan-Remo, Ogun State Nigeria; 3 S.C.C.D.P. Department, Microbiology and Clinical Microbiology Unit, University of Pavia, Pavia, Italy; 4 Dipartimento di Scienze e Tecnologie Alimentari per la Sostenibilità della Filiera Agro-Alimentare, Facoltà di Scienze Agrarie Alimentari ed Ambientali, Università Cattolica del Sacro Cuore, Via Emilia Parmense 84, Piacenza-Cremona, 29122 Italy; 5 I.R.C.C.S. Policlinico S. Matteo, Pavia, Italy; 6 Department of AgriFood Science, Institute of Advance Science for Agriculture, Jolanda di Savoia (FE), Italia

**Keywords:** *Enterobacter* spp., *Enterobacter hormaechei*, *E. cloacae*, Whole-genome sequencing, Sequence types, Drug resistance, Nigeria

## Abstract

Background: Extended-spectrum β-lactamases (ESBL) and AmpC β-lactamase-producing Enterobacter species are pathogens of public health concern. In this study, we investigated the resistome, virulome, and plasmidome of circulating Enterobacter hormaechei isolated from humans, animals, and ready-to-eat (RTE) food in Dutsin-Ma, northwest Nigeria. Materials and methods: Enterobacter species were isolated from human, and RTE food samples using MacConkey and CHROM agar. Species identification and antibiotics susceptibility testing (AST) were performed using Microscan autoSCAN-4 and MALDI-TOF MS, respectively. Whole-genome sequencing (WGS) and bioinformatics analysis were performed to validate the species identities. WGS also determined sequence types (STs), resistome, plasmidome, and virulome using PubMLST, ResFinder, PlasmidFinder, and Virulence Factors Database (VFDB) via ABRicate, respectively. Results: E. hormaechei subs. xiangfangensis was the dominant species isolated from humans (6/8, 75%) and animals (1/8, 12.5%), while E. hormaechei subs. oharae was exclusively found in RTE food (1/8, 12.5%). The most frequent sequence type (ST) was ST813, found solely in humans, along with ST511, ST454, and the emerging high-risk ST171. ST68 was identified in food, and novel ST (ST3186) were identified in animal and human samples, respectively. All isolates exhibited 100% resistance to ampicillin and amoxicillin-clavulanic acid, with 62.5% resistant to each of aztreonam, ceftazidime, ciprofloxacin, gentamicin, tobramycin and cefotaxime, and 25% to colistin. E. hormaechei (62.5%) isolates carried the blaCTX−M−15 gene, encoding ESBL-production, along with AmpC genes such as blaCMH−3, and also harbored resistance genes for aminoglycosides, fluoroquinolones, sulfonamides, tetracyclines, and trimethoprim. The IncHI2 (50%) was identified as the dominant plasmid type followed by Col (pHAD28) (25%), IncFIB (25%), and IncFII (11.1%). The most (62.5%) prevalence virulence gene among the isolates is ompA encoding adhesion while each of entA, entB and fepD encoding iron acquisition were least (12.5%) occurred. Conclusion: This study provides the first genomic insight into Enterobacter species in northwest Nigeria, highlighting their role in the spread of antimicrobial resistance. E. hormaechei subsp. xiangfangensis was identified as the main reservoir of ESBL and AmpC β-lactamases in this study. The discovery of novel STs with diverse virulence factors and plasmid replicon indicates potential horizontal gene transfer across different ecological niches. Our findings underscore the need for robust surveillance and control measures to combat the spread of MDR Enterobacter species across human, animal, and food niches.

## Introduction

The genus *Enterobacter* belongs to the *Enterobacteriaceae* family and includes motile, Gram-negative, facultative anaerobic, rod-shaped bacteria. Its taxonomy is complex and dynamic, with some species recently reclassified into other genera. *Enterobacter cloacae complex* (ECC) is a subgroup within the *Enterobacter* genus comprising multiple closely related species, including *Enterobacter cloacae* and others that are often difficult to distinguish using traditional biochemical tests. These opportunistic pathogens are common causes of foodborne illness and nosocomial infections, particularly in immunocompromised individuals (Davin-Regli and Pagès 2015, Wu et al. 2020, Davin-Regli et al. 2019).

The ECC subgroup includes seven species: *E. asburiae*,* E. cloacae*,* E. hormaechei*,* E. kobei*,* E. ludwigii*,* E. mori*, and *E. nimipressuralis*. Recently identified species, such as *E. roggenkampii*,* E. chengduensis*, and *E. bugandensis*, have also been associated with ECC (Doijad et al. 2016, Sutton et al. 2018, Wu et al. 2019). Hoffmann et al. reported that *Enterobacter hormaechei* is the predominant species among clinical isolates; however, many of these strains are often misidentified and incorrectly labeled as *E. cloacae* (Hoffmann et al. 2005). This taxonomic confusion arises from the incomplete genome sequencing of type strains and inconsistent naming conventions (Wu et al. 2020). Recent re-evaluations of the ECC have refined its phylogeny, yielding clearer boundaries between the constituent species and subspecies which revealed overlooked diversity (Sutton et al. 2018). The classification of *E. hormaechei* subspecies has progressed from relying on phenotypic assays and digital DNA–DNA hybridization (dDDH) to genome-based average nucleotide identity (ANI) analysis, revealing previously overlooked diversity (Sutton et al. 2018). Phylogenomic analysis showed that *E. hormaechei* is a complex species consisting of three predicted species *E. hormaechei*, *E. hoffmannii*, and *E. xiangfangensis*. The last of these, *E. xiangfangensis*, is thought to include three distinct subspecies: *oharae*, *steigerwaltii*, and *xiangfangensis* (Maayer et al. 2025). These advances underscore the increasingly central role of computational genome analysis in contemporary bacterial systematics.

Owing to its evolving multidrug resistance, *Enterobacter* spp. has been included in the World Health Organization (WHO) priority pathogen list critical for research and discovery, which consists of *Escherichia coli*,* Staphylococcus aureus*,* Klebsiella pneumoniae*,* Acinetobacter baumannii*,* Pseudomonas aeruginosa*,* Enterobacter*, and *Enterococcus* species (collectively known as ESKAPEE). These pathogens are known for their ability to “escape” the effects of antimicrobial agents and are responsible for a wide range of both opportunistic and hospital-acquired infections (Maayer et al. 2025, Álvarez-Marín et al. 2021, World Health Organization 2024). Recently, *E. hormaechei* have been implicated in the contamination of ready-to-eat fruits and food in Nigeria, as well as in outbreaks of foodborne illness in Mexico (Makinde et al. 2021, Oyedele et al. 2020, Parra-Flores et al. 2018). Species within the ECC subgroup have increasingly emerged as pathogens of public health concern due to the widespread antibiotic resistance observed among multidrug-resistant strains. These factors have contributed to the development of a broad, relatively conserved set of pathogenicity determinants, and a variable array of secretion systems (Maayer et al. 2025). These bacteria are inherently resistant to β-lactam antibiotics, including drugs commonly used in clinical practice, such as penicillins, and first-generation cephalosporins (Davin-Regli and Pagès 2015, Wu et al. 2018, Zhu et al. 2017, Chavda et al. 2016).

Due to the variability in antimicrobial resistance pattern, virulence, and dissemination potential, accurate identification of different ECC with relevant features of clinical and public health concern at both the species and subspecies levels is crucial for a thorough understanding of their epidemiology, pathogenesis, and microbiological characteristics (Annavajhala et al. 2019, Xu et al. 2022, Qiu et al. 2024). We hypothesize that there is significant variation in antimicrobial resistance patterns, virulence factors, and clonal dissemination of *Enterobacter* species circulating in the north-western region of Nigeria, which may be potentially linked to regional factors such as healthcare practices, antibiotic use in food-producing animals, and environmental contamination. Additionally, various virulence genes have been identified in *Enterobacter* spp., including those responsible for different adhesins, biofilm formation, iron acquisition systems, stress responses, and several genes associated with toxin secretion systems (Liu et al. 2022, Ganbold et al. 2023).

Given the significance of ECC as emerging public health pathogens, often resistant to antimicrobial agents and, in severe cases, acquiring antimicrobial resistance (Uchida et al. 2020), genomic surveillance of the pathogens is essential for understanding their evolution and epidemiology. However, there is limited literature on the complete genome of ECC isolated in northern Nigeria. To fill this gap, we analyzed whole-genome datasets to report the resistomes of *E. hormaechei* isolated from human, and ready-to-eat-food samples to elucidate their dissemination patterns within the Dutsin-Ma metropolis in northwest, Nigeria.

## Materials and methods

### Sampling location, sample collection, and bacterial isolation

This study was a continuation of our previous studies (Adesoji et al. 2025, Adesoji et al. 2025b) conducted in Dutsin-Ma, Katsina State, Nigeria, located at 12°27’18’’N, 7°29’29’’E, with a population of 169,671 and an area of 527 km² as of 2006 (Jain et al. 2018). The town is predominantly inhabited by the Hausa and Fulani communities, who are engaging in farming, animal husbandry, and trading. It is also situated near Zobe Dam and serves as the host community for the Federal University Dutsin-Ma. Samples including food (ready-to-eat foods, tubers, fruits, and vegetables), raw meat (cow and goat meat), and human clinical (urine and fecal) samples were collected between October and November 2022. Food samples were obtained from the local market, while clinical samples were collected from Malam Mande General Hospital and the Comprehensive Health Care Centre. A total of 300 samples (180 food and 120 clinical) were collected and transported to the Microbiology Laboratory at the Federal University Dutsin-Ma for bacterial isolation.

For bacterial isolation, samples were plated on MacConkey agar. Colonies appearing pink mucoid on MacConkey agar were selected and subcultured to obtain pure cultures. These were separately stored on double-strength nutrient agar slants at 4 °C and shipped to the University of Pavia, Italy. There, isolates were re-streaked on chromogenic agar, and presumptive *Enterobacter* species were selected based on metallic blue appearance (Adesoji et al. 2025).

### Bacterial identification and antimicrobial susceptibility testing

Preliminary species identification and antimicrobial susceptibility testing (AST) was carried out using the semi-automated system MicroScan autoSCAN-4 (Beckman Coulter). Additional species confirmation was assessed with Matrix Assisted Laser Desorption/Ionisation Time-Of-Flight Mass Spectrometry (MALDI-TOF MS; Bruker Daltonics, Bremen, Germany) equipped with Bruker Biotyper 3.1 software. Minimum inhibitory concentration (MIC) values were interpreted according to the European Committee on Antimicrobial Susceptibility Testing (EUCAST version 14, 2024) breakpoints (The European Committee on Antimicrobial Susceptibility Testing. Breakpoint tables for interpretation of MICs and zone diameters http://www.eucast.org*).* The tested antibiotics included amikacin (AMK), amoxicillin/clavulanic acid (AMC), ampicillin (AMP), aztreonam (AZT), cefepime (CFP), cefoxatime (CFT), ceftazidime-avibactam (CZA), ceftazidime (CTZ), ciprofloxacin (CIP), colistin (COL), ertapenem (ERT), gentamicin (GEN), levofloxacin (LEV), meropenem (MEP), piperacillin-tazobactam (PIT), tobramycin (TOB), trimethoprim/sulfamethoxazole (STX).

### Whole-genome sequencing (WGS) and genome analyses

Eight strains underwent Whole-Genome Sequencing (WGS). The genomic DNA were extracted using the DNeasy Blood & Tissue kit (Qiagen) and was sequenced on an Illumina HiSeq PE150 platform (Illumina Inc., San Diego, CA, USA) with a 350-bp paired-end sequencing, after libraries preparation with Nextera XT library preparation kit. Read quality check was assessed with FastQC. Reads were assembled within the Shovill pipeline, discharging short contigs, with low coverage, or pure homopolymers (T. Seeman, https://github.com/tseemann/shovill*).*

Assembled sequences were annotated using Prokka and manually checked by Rapid Annotation using Subsystems Technology (RAST server). Species identification was assessed using the online tool Type (Strain) Genome Server (https://tygs.dsmz.de/), and performing the average nucleotide identity (ANI) analysis using the FastANI tool (Jain et al. 2018), and the digital DNA-DNA hybridization (dDDH) values using formula 2 of Genome-to-Genome Distance Calculator version 2.1 (Meier-Kolthoff et al. 2013). Resistome, plasmidome and virulome were determined through ResFinder, PlasmidFinder and the Virulence Factors Database (VFDB) via ABRicate (github.com/tseemann/ABRicate). In silico multi-locus sequence typing (MLST) was assigned through *Enterobacter* spp. via PubMLST database (Jolley et al. 2018).

### SNP analysis and phylogenetic reconstruction

Core genome and single nucleotide polymorphisms (SNPs) phylogenetic relationships were investigated. Specific dataset for each sequence type (ST) was built by downloading available genomes from PubMLST (https://pubmlst.org/organisms/enterobacter-cloacae). In detail, a total of 30 public *E. hormaechei* genomes were retrieved for ST171, nine genomes for ST511, and three each for ST68 and ST813, respectively, while one was retrieved for ST454. SNPs-based phylogenies were obtained using the online tool CSI Phylogeny 1.4, available on the Center for Genomic Epidemiology (https://cge.food.dtu.dk/*)* (Kaas et al. 2014). Graphic illustrations of the trees were built with the interactive tree of life (iTOL) (https://itol.embl.de/). Pairwise SNPs between strains were counted from the core SNPs alignment with snp-dists 0.7.0 (https://github.com/tseemann/snp-dists*).*

### Graphical representation

Graphs were drawn using GraphPad Prism version 10.3.1 (464) for macOS, GraphPad Software, Boston, Massachusetts USA, www.graphpad.com.

## Results

### Samples origin and species identification

In the period October-November 2022, from the analysis of 300 human, food and animal samples, eight *Enterobacter* spp. strains were isolated. Of these, six were obtained from human samples (five from urine and one from feces), while the remaining two were isolated from food and animal samples, respectively. Regarding the six strains of human origin, two out of six were obtained from the Mallam Mande general hospital and the remaining four from the Comprehensive Health Center, both located in Dutsin-Ma town.

In accordance with MicroSCAN results, all eight isolates were identified as *Enterobacter cloacae*. In contrast, the MALDI-TOF identification pointed out five *E. cloacae*, two *Enterobacter xiangfangensis* and one *Enterobacter bugandensis*. A deeper investigation through the genome analysis revealed that six strains, five of human origin and one of animal origin, belonged to *E. hormaechei* subs. *xiangfangensis*. The other two strains, one isolated from human and the other isolated from food, were identified as *E. cloacae* and *E. hormaechei* subs. *oharae*, respectively (Table 1).

**Table 1:** Species identification

Strains	Sources	Date of collection	Species (MicroScan AutoSCAN4)	Species (MALDI-TOF)	Species (FastANI)	dDDH^2^ ^(% ID)^
FC113	Animal	23th November 2022	*E. cloacae*	*E. cloacae*	*E. hormaechei* subs. *xiangfangensis*	99.33
U28	Human	27th October 2022	*E. cloacae*	*E. cloacae*	*E. hormaechei* subs. *xiangfangensis*	99.33
U29	Human	27th October 2022	*E. cloacae*	*E. cloacae*	*E. hormaechei* subs. *xiangfangensis*	99.26
U176	Human	25th November 2022	*E. cloacae*	*E. cloacae*	*E. hormaechei* subs. *xiangfangensis*	99.35
U184C	Human	25th November 2022	*E. cloacae*	*E. cloacae*	*E. hormaechei* subs. *xiangfangensis*	99.32
F59	Food	3rd November 2022	*E. cloacae*	*E*. *xiangfangensis*	*E. hormaechei* subs. *oharae*	98.53
FC203B2	Human	29th November 2022	*E. cloacae*	*E. bugandensis*	*E. cloacae*	98.87
U30	Human	27th October 2022	*E. cloacae*	*E*. *xiangfangensis*	*E. hormaechei* subs. *xiangfangensis*	99.32

### Antimicrobial resistance characterization

**Fig. 1:**
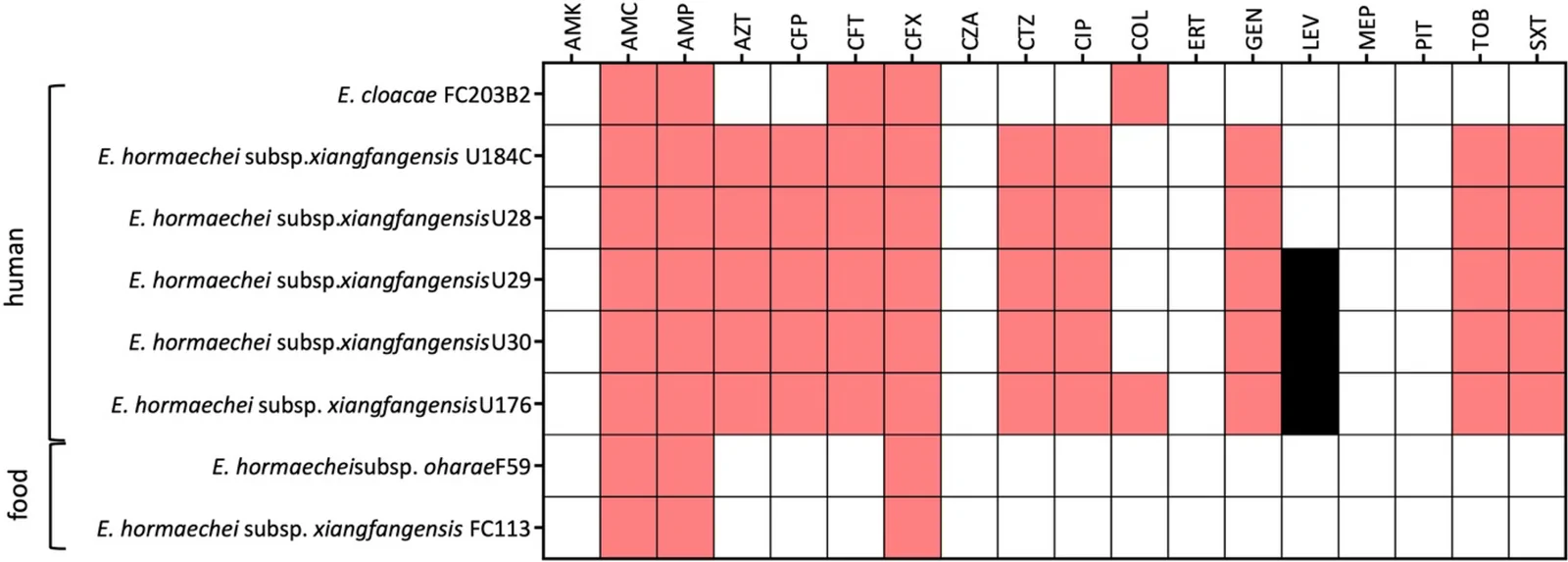
Phenotypic resistance pattern of the eight *Enterobacter* spp. strains isolated from this study. Color code indicates the resistant, sensitive or intermediate phenotype. AMK: amikacin, AMC: Amoxicillin/clavulanic acid, AMP: ampicillin, AZT: aztreonam, CFP: cefepime, CFT: cefoxatime, CZA: ceftazidime-avibactam, CTZ: ceftazidime, CIP: ciprofloxacin, COL: colistin, ERT: ertapenem, GEN: gentamicin, LEV: levofloxacin, MEP: meropenem, PIT: piperacillin-tazobactam, TOB: tobramicin, STX: trimethoprim/sulfamethoxazole.

Distinct resistance patterns were observed across *Enetrobacter* isolates, with notable differences between human- and food-derived strains (Fig. 1).

Food- and animal-derived isolates (F59 and FC113) exhibited relatively limited resistance phenotypes. Resistance was primarily observed against amoxicillin/clavulanic acid, ampicillin and ciprofloxacin, whereas they remained susceptible to carbapenems (ertapenem and meropenem), colistin, aminoglycosides and fluoroquinolones. In contrast, clinical isolates (U184C, U28, U29, U30, and U176) demonstrated markedly broader resistance profiles. All clinical isolates were resistant to cefotaxime (third-generation cephalosporins), with elevated MIC values indicative of extended-spectrum β-lactamase (ESBL) production. All human isolates, except FC203B2, were resistant also to aztreonam and ceftazidime. The same strains were found to exceed the EUCAST cutoff for ciprofloxacin, gentamycin and tobramycin. Only FC203B2 and U176 were found to be resistant to colistin. On the contrary, they showed a sensitive phenotype to amikacin, ceftazidime-avibactam and carbapenems, such as ertapenem and meropenem. Based on the antibiogram, five strains exhibited a multi-drug resistant (MDR) phenotype, showing resistance to β-lactams (ampicillin, third- and fourth-generation cephalosporins), aminoglycoside (gentamycin, tobramycin) and fluoroquinolones (ciprofloxacin).

### WGS analysis

**Fig. 2:**
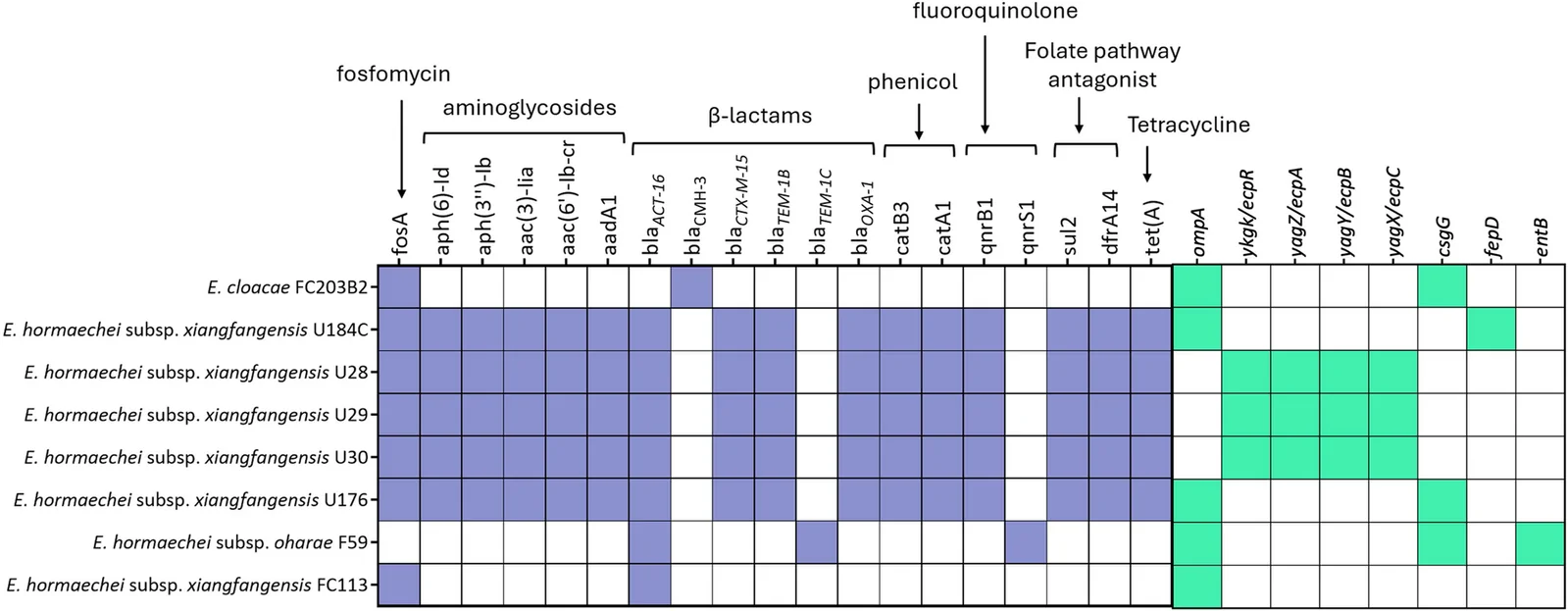
Distribution of antimicrobial resistance and virulence genes among *Enterobacter* spp. strains isolated from human and food. Mauve color represents the positivity of the strain for each gene. The expected phenotypic resistance has been indicated. The green color identifies the presence of virulence markers

Concerning the AMR gene contents, the eight *Enterobacter* strains exhibited a diverse resistome (Fig. 2). All isolates, with the exception of the food-derived *E. hormaechei* subsp. *oharae* F59, harbored the *fosA* gene, which confers resistance to fosfomycin. Notably, the five human-derived isolates *E. hormaechei* subs. *xiangfangensis* U184C, U28, U29, U30 and U176 shared and identical ARGs profile. This included multiple genes associated with aminoglycoside resistance (*aph(6)-Id*, *aph(3’’)-Ib*, *aac(3)-IIa*, *aac(6’)-Ib-cr*, *aadA1*), phenicol resistance (*catA1*, *catB3*), sulfonamide resistance (*sul2*), fluoroquinolone resistance (*qnrS1*), tetracycline resistance (*tet(A)*), and trimethoprim resistance (*dfrA14*). In addition, these isolates carried several β-lactamase genes, including *bla*_ACT−16_, *bla*_CTX−M−15_, *bla*_TEM−1B_, and *bla*_OXA−1_, indicating a broad resistance to β-lactam antibiotics (Fig. 2).

In contrast, the remaining human-derived isolate, FC203B2 (*E. cloacae*), displayed a more limited resistome. Besides *fosA*, it carried only *bla*_CMH−3_, a gene encoding an AmpC β-lactamase associated with resistance to extended-spectrum β-lactams. Compared to human isolates, food-derived strains exhibited a less extensive repertoire of AMR genes. The *E. hormaechei* subsp. *oharae* F59 isolate carried *bla*_ACT−16_, as observed in most other strains except *E. cloacae* FC203B2. Additionally, it harbored *bla*_TEM−1 C_ and *qnrS*, distinguishing it from the other isolates. The second food-derived isolate, FC113, showed the most limited resistome, carrying only *fosA* and *bla*_ACT−16_. No acquired AMR genes for carbapenems or colistin were detected (Table 2).

**Table 2:** Whole-genome sequencing results

Strains	Sources	Species	STs	Resistome	Virulome	Plasmidome
FC113	Animal	*E. hormaechei* subs. *xiangfangensis*	3186	*bla*ACT-16, *fosA*	*ompA*	ND
U28	Human	*E. hormaechei* subs. *xiangfangensis*	813	*aph(6)-Id*, *aph(3’’)-Ib*, *aac(3)-Iia*, *aac(6’)-Ib-cr*, *aadA1*, *bla*_ACT−16,_*bla*_CTX−M−15_, *bla*_TEM−1B_, *bla*_OXA−1_, *fosA*, *catB3*, *catA1*, *qnrB1*, *sul2*, *tet(A)*, *dfrA14*	*yagX/ecpC*,* yagY/ecpB*,* yagZ/ecpA*,* ykgk/ecpR*	IncHI2, IncHI2A
U29	Human	*E. hormaechei* subs. *xiangfangensis*	813	*aph(6)-Id*, *aph(3’’)-Ib*, *aac(3)-Iia*, *aac(6’)-Ib-cr*, *aadA1*, *bla*_ACT−16_, *bla*_CTX−M−15_, *bla*_TEM−1B_, *bla*_OXA−1_, *fosA*, *catB3*, *catA1*, *qnrB1*, *sul2*, *tet(A)*, *dfrA14*	*yagX/ecpC*,* yagY/ecpB*,* yagZ/ecpA*,* ykgk/ecpR*	IncHI2, IncHI2A
U176	Human	*E. hormaechei* subs. *xiangfangensis*	511	*aph(6)-Id*, *aph(3’’)-Ib*, *aac(3)-Iia*, *aac(6’)-Ib-cr*, *aadA1*, *bla*_ACT−16,_, *bla*_CTX−M−15_, *bla*_TEM−1B_, *bla*_OXA−1_, *fosA*, *catB3*, *catA1*, *qnrB1*, *sul2*, *tet(A)*, *dfrA14*	*csgG*,* ompA*	IncHI2, IncHI2A
U184C	Human	*E. hormaechei* subs. *xiangfangensis*	171	*aph(6)-Id*, *aph(3’’)-Ib*, *aac(3)-Iia*, *aac(6’)-Ib-cr*, *aadA1*, *bla*_ACT−16,_, *bla*_CTX−M−15_, *bla*_TEM−1B_, *bla*_OXA−1_, *fosA*, *catB3*, *catA1*, *qnrB1*, *sul2*, *tet(A)*, *dfrA14*	*fepD*,* ompA*	Col(pHAD28), IncFIB(pECLA), IncFII(pECLA)
F59	Food	*E. hormaechei* subs. *oharae*	68	*bla*_ACT−16_, *bla*_TEM−1 C_, *qnrS1*	*csgG*,* entB*,* ompA*	Col(pHAD28), IncFIB(k), IncFII(Yp), IncX10
FC203B2	Human	*E. cloacae*	454	*bla*_CMH−3_, *fosA*	*csgG*,* ompA*	No plasmid
U30	Human	*E. hormaechei* subs. *xiangfangensis*	813	*aph(6)-Id*, *aph(3’’)-Ib*, *aac(3)-Iia*, *aac(6’)-Ib-cr*, *aadA1*, *bla*_ACT−16,_, *bla*_CTX−M−15_, *bla*_TEM−1B_, *bla*_OXA−1_, *fosA*, *catB3*, *catA1*, *qnrB1*, *sul2*, *tet(A)*, *dfrA14*	*yagX/ecpC*,* yagY/ecpB*,* yagZ/ecpA*,* ykgk/ecpR*	IncHI2, IncHI2A

Regarding the plasmidome, no plasmids were detected in the food-derived *E. hormaechei* subsp. *xiangfangensis* strain FC113 or in the human-derived *E. cloacae* strain FC203B2. Four *E. hormaechei* subsp. *xiangfangensis* strains (U28, U29, U30, and U176) shared the same combination of replicon types (IncHI2 and IncHI2A). In contrast, strain U184C of *E. hormaechei* subsp. *xiangfangensis* harbored Col(pHAD28), IncFIB(pECLA), and IncFII(pECLA), while the food-derived *E. hormaechei* subsp. *oharae* strain carried Col(pHAD28), IncFIB(k), IncFII(Yp), and IncX10.

The sequence types based on the PubMLST scheme, revealed a polyclonal heterogeneity of STs. The ST813 was the predominant ST, associated exclusively with human niche as well as ST511, ST171 and ST454. The only occurrence of ST68 was associated with the food niches. Additionally, one novel sequence type was identified: ST3186, that was assigned to *E. hormaechei* (strain FC113) of animal origin.

The overall virulome was narrow (Fig. 2). Five isolates, namely *E. cloacae* FC203B2, *E. hormaechei* subs. *xiangfangensis* U184C, U176, FC113 and *E. hormaechei* subsp. *oharae* F59 carried the outer membrane protein gene *ompA*. The other three human-derived *E. hormaechei* subs. *xiangfangensis* lacked *ompA* but harbored genes encoding the *E. coli* common pilus (ECP) adhesion system, including *yagX/ecpC*,* yagY/ecpB*,* yagZ/ecpA*,* ykgk/ecpR.* Moreover, adhesion and colonization system gene as *csg* was detected in FC203B2, U176 and F59. Lastly, *fepD* and *entB* genes associated with siderophore-mediated iron uptake pathway genes were found in U184C and F59, respectively.

### Phylogenetic analysis

#### *E. hormaechei* ST171

**Fig. 3:**
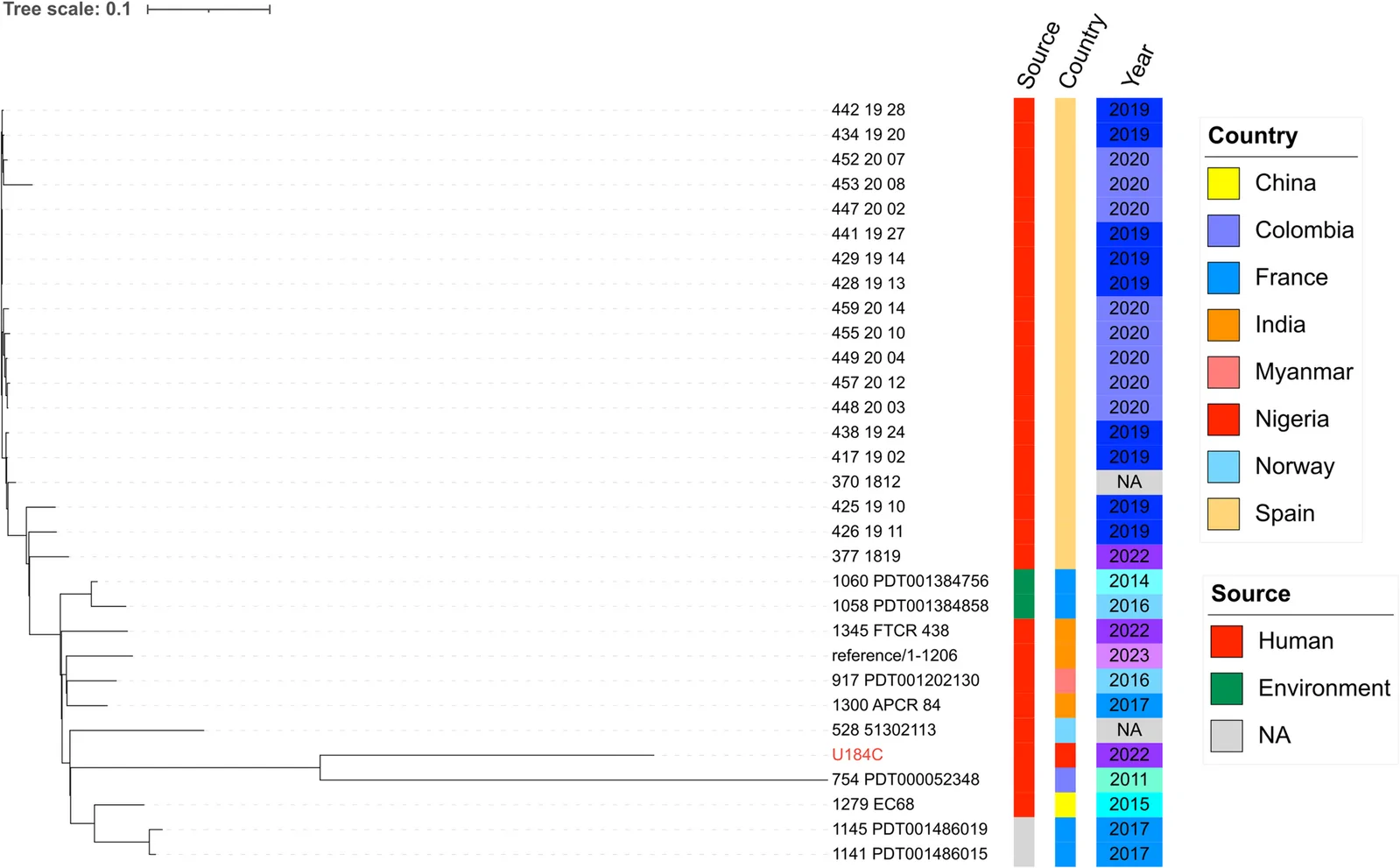
SNP-based phylogeny pictured using iTOL v6 of the available *E. hormaechei* ST171

The U184C was compared with the available genomes for *E. hormaechei* ST171 (*n* = 30), retrieved from PubMLST database. Based on the available metadata, ST171 clones circulated predominantly in European regions (Spain, France and Norway), and sporadically in Asia, while they were absent in the African continent. The U184C appeared to be genomically unique clone, clustering with PDT000052348 (number of SNPs > 400), which was collected in Colombia in 2011 (Fig. 3). These data suggest the emergence of *E. hormaechei* ST171 in the human niche in Nigeria.

#### Minor STs

**Fig. 4:**
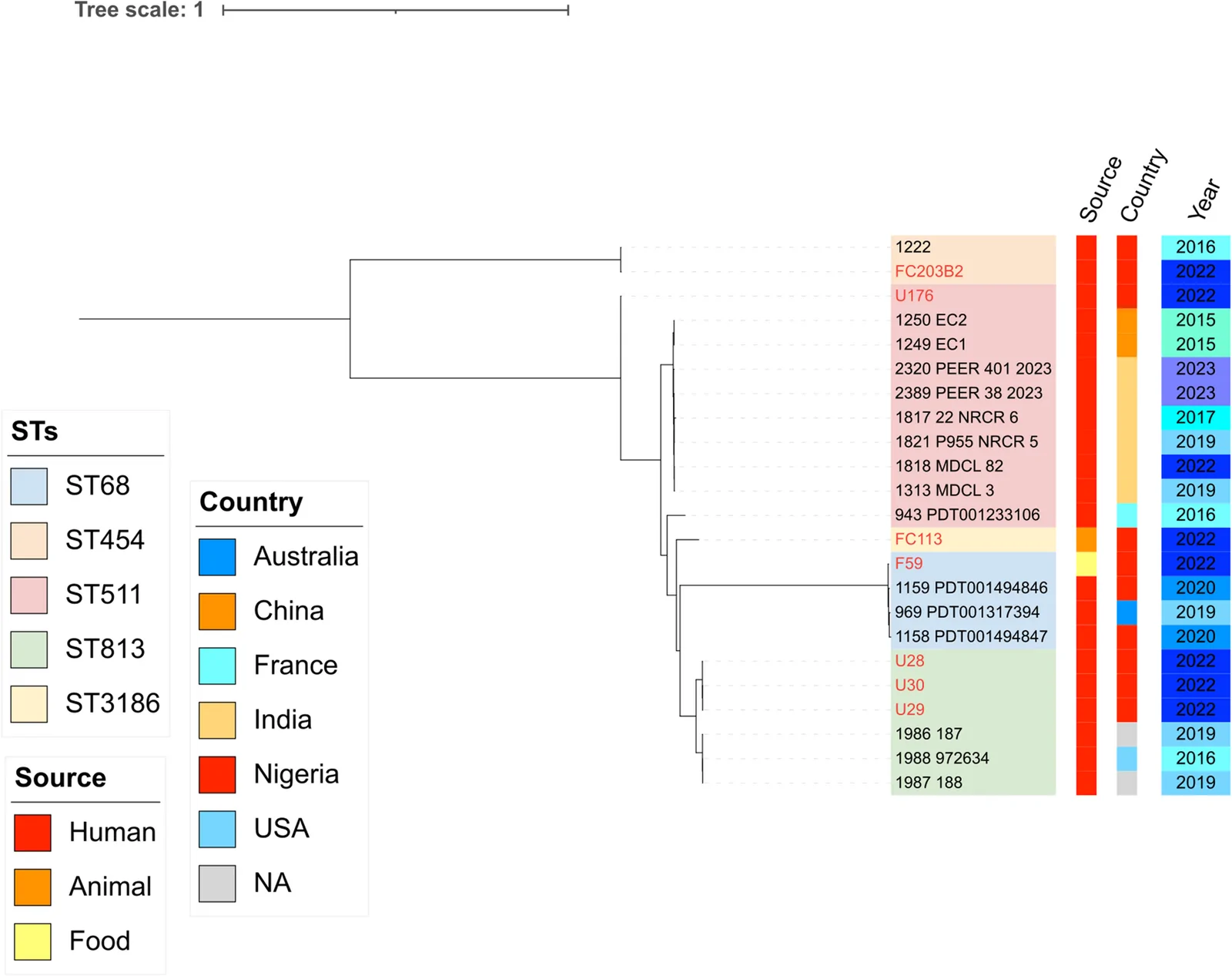
SNP-based phylogeny pictured using iTOL v6 of the available *E. hormaechei* ST68, ST454, ST511 and ST813

Based on the available metadata, *E. hormaechei* ST511 has a narrow diffusion range, with genomes identified only in China, India and France. The Nigerian strain U176 was clonally distant from other ST511 strains, exhibiting lowest number of SNPs (5,500) with “1821” (Fig. 4). Regarding the ST454, the two available genomes are from human Nigerian niche, collected in 2016 and 2022, respectively. SNPs-based phylogeny highlighted a clonal relationship between the two genomes, with only 61 SNPs differences. These data speculate a Nigerian origin for the ST454 clone and its stable presence since 2016. Similarly, the ST68 appears to circulate exclusively in Nigeria and Australia (Fig. 4). Despite F59 was isolated from food sources, ST68 seemed more closely associated with human environments. The SNPs evaluation revealed a moderate level of clonal relatedness (number of SNPs < 350). Interestingly, F59 exhibited stronger similarity to the Australian strain “969” (number of SNPs = 256) and the Nigerian “1159” (number of SNPs = 194) strain. Based on the collection timeline, these data support the hypothesis of the entry of this clone into Nigeria from Australia. Moreover, the detection of these clones in both human and food niches highlights the connection between the two environments in facilitating their spread within Nigeria. For the ST813, the SNPs-based phylogeny identified two clusters: one Nigerian, comprising U28, U29 and U30 and an unknown cluster including strains “1986”, “1987,” and “1988”. The two clusters were clonally distant, with 1,460-1,439 SNPs differences. The members of the Nigerian cluster were highly related (number of SNPs < 29), with U29 and U30 differing by only three SNPs. Together, these findings revealed the emergence of heterogeneous clones in different niches in Nigeria.

## Discussion

The ESKAPEE pathogens represent a major threat to global health due to their capacity to evade antimicrobial activity and drive multidrug-resistant infections. Among them, *Enterobacter cloacae* complex (ECC), encompassing different species, is increasingly recognized as important opportunistic pathogens responsible for a wide range of healthcare-associated infections, including bloodstream infections, particularly in vulnerable and hospitalized populations (Miller and Arias 2024). These microbes are present in many different niches, including natural environment, water, food and animals, representing a global issue to be faced with a One Health perspective.

An accurate species-level identification remains challenging in routine clinical microbiology, as conventional biochemical and automated systems frequently misclassify isolates (Kaplan et al. 2025). Here, the preliminary analysis with MALDI-TOF identified all the strains as *E. hormaechei*, but the following genome analysis based on DDH and ANI, allowed to distinguish them into *E. hormaechei* subsp. *xiangfangensis* (six strains), *E. hormaechei* subsp. *oharae* (one strain) and *E. cloacae* (one strain). Although the MALDI-TOF MS Biotyper is widely regarded as a reliable method with good discriminatory power, our study indicated that WGS coupled with bioinformatic analysis may offer additional resolution for species-level identification, accordingly with other previous studies (Kluytmans-van den Bergh et al. 2016, Oni et al. 2025).

The *Enterobacter cloacae* complex (ECC) is characterized by a highly plastic genome, which facilitates the acquisition and exchange of genetic material via horizontal gene transfer and enables adaptation to diverse ecological niches (Oni et al. 2025, Liu et al. 2013, Maguvu and Bezuidenhout 2021). This genomic flexibility promotes the dissemination of ARGs, posing a significant public health challenge, particularly in low- and middle-income countries where therapeutic options are limited (Wang et al. 2025). From 300 samples of human and food origin, eight *Enterobacter* spp. isolates were selected as resistant to many antimicrobials and five showed an MDR phenotype. The analysis of antimicrobial susceptibility profiles showed a distinction between human-derived and food-derived isolates. Clinical strains belong to *E. hormaechei* subs. *xiangfangensis* species exhibited broader resistance patterns, with most isolates displaying MDR phenotypes and resistance to third-generation cephalosporins, fluoroquinolones, and aminoglycosides. This is in line with global reports describing ECC as a major reservoir of β-lactamases and other resistance determinants in hospital environments, worldwide and in African countries. Sumbana et al. reported the characterization of eight strains of *E. cloacae* comples strains isolated from bloodstream infections in Mozambique, highlighting the prevalence of ESBL phenotype associated with the resistance to trimethoprim–sulfamethoxazole, gentamicin, ciprofloxacin, fosfomycin, colistin. Moreover, a large investigation in seven low- and middle-income countries indicated the resistance to a wide range of antimicrobials, with high prelavence of ESBL strains, as found also by others (Sands et al. 2021, Jesumirhewe et al. 2020). The other strains were isolated from food producing animal origin and displayed a limited AMR profile. Besides humans, the presence of MDR ECC was found also in food and animals although limited efforts were implied so far to investigate the circulation of this microbial group in non-human niches (Yossapol et al. 2017, Haenni et al. 2016, Esteban-Cuesta et al. 2019). These results suggest a lower selective pressure in non-clinical environments, although they may still act as reservoir of ARGs, contributing to their spread to humans.

Both ESBL and broad spectrum β-lactamase phenotype found in six isolates was confirmed at genome level with an extensive repertoire of ARGs including *bla*_CTX−M−15_, *bla*_TEM−1B_ and *bla*_OXA−1_. The co-occurrence of multiple ARGs conferring resistance to aminoglycosides, fluoroquinolones, sulfonamides, and tetracyclines further explains the MDR phenotype observed. The co-occurrence of ESBL genes and aminoglycosides and quinolone resistance was often observed (Annavajhala et al. 2019). Although two strains showed the resistance to colistin, a related genetic determinant was not found.

Virulence factors have garnered significant attention because of their critical role in the pathogenesis of bacterial species. Five strains carried *ompA*, which encodes a highly conserved outer membrane protein in the *Enterobacteriaceae* family, which is important for membrane integrity, eukaryotic cell infectivity, immunomodulation, and mediates the adhesion and invasion of epithelial cells and macrophages (March et al. 2011). The genes *yagX/ecpC*,* yagY/ecpB*,* yagZ/ecpA*, and *ykgK/ecpR* were exclusively found in *E. hormaechei* subs. *xiangfangensis*. These genes encode common pili structural subunits in *Enterobacteriaceae* (Ievy et al. 2022) and are involved in the adhesion and invasion of bacteria into host cells. Moreover, the detection of the curli-encoding gene *csgG* in the genomes of *E. hormaechei* strains further highlight their capacity to adhere to and invade host cells (Rahi et al. 2024). Furthermore, siderophore biosynthesis genes, including *fepD*, *entA*, and *entB*, are among the detected virulence repertoires and play a vital role in iron acquisition by facilitating the scavenging of iron from the host through specialized iron-transport systems. Similar findings have been documented in studies examining the resistome and virulome of ECC (Folgori et al. 2021, Bolourchi et al. 2022). Research on virulence factors remains limited; therefore, further studies are needed to better characterize the genes involved in pathogenesis mechanisms, including the ability to form biofilm and persist in the environment.

The ST171 is an emerging high-risk clone associated with bloodstream infection and the production of relevant antimicrobial-resistant enzymes, such as OXA-48 and *mcr*-like (Lumbreras-Iglesias et al. 2023). The emergence of carbapenemases-producing *E. hormaechei* ST171 has been reported in both human and environmental contexts in Spain, and the USA (Lumbreras-Iglesias et al. 2023, Ballash et al. 2023, Ghazawi et al. 2024). Moreover, the ST171 clone has been reported to be associated with relevant virulence factors, including toxin production and biofilm formation (Ghazawi et al. 2024).

*Enterobacter hormaechei* has been reported as contaminants in vended fresh-cut fruits and ready-to-eat foods in southwest Nigeria (Makinde et al. 2021, Oyedele et al. 2020). Similarly, this study reveals the contamination of food by *Enterobacter* species in northwest Nigeria. These findings suggest fecal contamination of food or water, posing significant public health risks to consumers. Approximately 600 million people fall ill worldwide (nearly 1 in 10 people) from consuming contaminated food, with 420,000 deaths annually. This results in a loss of 33 million healthy life years, measured in Disability-Adjusted Life Years (DALYs), based on estimates from 2015 data (Kim et al. 2018).

More than half of the *E. hormaechei* isolates (55.5%) characterized in this study harbored *bla*_CTX−M_. Similar results have been documented in France, Guadeloupe, and Taiwan, where *E. hormaechei* exhibited higher resistance rates to third-generation cephalosporins than other ECC members (Garinet et al. 2018, Pot et al. 2021, Chang et al. 2022). Additionally, the ESBL-producing isolates exhibited resistance to various antimicrobial classes other than β-lactams, including aminoglycosides, fluoroquinolones, tetracyclines, chloramphenicol, fosfomycin, and trimethoprim/sulfamethoxazole. A similar observation has been documented in France (Haenni et al. 2016). These findings highlight the global dissemination of ESBL-producing *E. hormaechei* carrying *bla*_*CTX−M*_. and *Ampc*-β-lacta*m*ses, emphasizing their significant role in antimicrobial resistance, particularly against third-generation cephalosporins.

Surveys indicate that food-producing animals are responsible for transmitting over 70% of foodborne diseases (Focker and Fels-Klerx 2020). This is consistent with our finding in this current study that a novel sequence type (ST3186) of *E. hormaechei* was identified in food-producing animal sample. Similary, *E. hormaechei*, which produces the carbapenemase OXA-48, has been repeatedly identified in infections among companion animals hospitalized at a Swiss veterinary clinic. Some of these strains are clonally related to the animal and human ST114 strains (Donà et al. 2023). These findings highlight the potential risk of zoonotic dissemination of the novel ST3186 *E. hormaechei* strain.

IncHI2 and IncHI2A were the predominant plasmid types identified in the present study. ESBLs and carbapenemase-producing (CP) genes in *E. hormaechei* are commonly associated with plasmids, with the IncHI2 family being frequently documented (Sadek et al. 2020, Macesic et al. 2021, Algarni et al. 2024). The identification of several well-characterized *Inc* group plasmids in *E. hormaechei* isolates in this study underscores the ability of the species to effectively acquire and disseminate plasmids associated with the spread of critical ARGs.

The primary limitation of this study is the small number of *Enterobacter* isolates analyzed. Consequently, these findings may not fully capture the complete genetic diversity, clonal structure, or true prevalence of *Enterobacter* species circulating across human, animal, and food niches in northwest Nigeria. While our WGS approach provided detailed, high-resolution insights into specific high-risk clones (e.g., ST171), resistance mechanisms (*bla*_CTX−M15_ and novel sequence types (ST3186), caution must be exercised when extrapolating these results to broader regional or national populations. Future prospective studies incorporating larger sample sizes and broader geographical sampling are required to comprehensively map the transmission dynamics and epidemiology of multidrug-resistant *Enterobacter* species in Northern Nigeria.

## Conclusion

To our knowledge, this is the first genomic study to provide insights into the epidemiology of *Enterobacter* species in northwest Nigeria, revealing their significant role in the spread of AMR. Our findings highlight the dominance of *Enterobacter hormaechei* subs. *xiangfangensis* as reservoirs of ESBL and AmpC β-lactamases, with a concerning pattern of multidrug resistance. The identification of novel sequence types with diverse virulence profiles and plasmidomes further underscores the potential for horizontal gene transfer of relevant ARGs across various ecological niches. These findings indicate the emergence of MDR novel STs and emphasize the need for urgent, comprehensive surveillance and targeted interventions to enhance infection control measures, promote prudent antimicrobial stewardship, and mitigate this public health threat in humans, animals, and the food supply chain in northern Nigeria.

## Data Availability

The nucleotide sequences of the nine genomes were deposited and are available in GenBank under the Bioproject ID PRJNA1135675.

## References

[CR1] Davin-Regli A, Pagès JM (2015) *Enterobacter aerogenes* and *Enterobacter cloacae*; versatile bacterial pathogens confronting antibiotic treatment. Front Microbiol 6:392. 10.3389/fmicb.2015.00392 PMC4435039 26042091

[CR2] Wu W, Feng Y, Zong Z (2020) Precise species identification for Enterobacter: a genome sequence-based study with reporting of two novel species, Enterobacter quasiroggenkampii sp. nov. and Enterobacter quasimori sp. nov. MSystems 5(4):10–1128. 10.1128/msystems.00527-20 PMC7406230 32753511

[CR3] Davin-Regli A, Lavigne J-P, Pages J-M (2019) Enterobacter spp.: Update on taxonomy, clinical aspects, and emerging antimicrobial resistance. Clin Microbiol Rev 32(4):e00002–19. 10.1128/CMR PMC6750132 31315895

[CR4] Doijad S, Imirzalioglu C, Yao Y, Pati NB, Falgenhauer L, Hain T et al (2016) *Enterobacter bugandensis* sp. *nov*., isolated from neonatal blood. Int J Syst Evol Microbiol 66:968–974. 10.1099/ijsem.0.000821 26637818

[CR5] Sutton GG, Brinkac LM, Clarke TH, Fouts DE (2018) Enterobacter hormaechei subsp. hoffmannii subsp. nov., Enterobacter hormaechei subsp. xiangfangensis comb. nov., Enterobacter roggenkampii sp. nov., and Enterobacter muelleri is a later heterotypic synonym of Enterobacter asburiae based on computational analysis of sequenced Enterobacter genomes. F1000Res 7:521. 10.12688/f1000research.14566.2 PMC6097438 30430006

[CR6] Wu W, Feng Y, Zong Z (2019) Characterization of a strain representing a new *Enterobacter* species, *Enterobacter chengduensis* sp. nov. Antonie Van Leeuwenhoek 112:491–500. 10.1007/s10482-018-1180-z 30302649

[CR7] Hoffmann H, Schmoldt S, Trülzsch K, Stumpf A, Bengsch S, Blankenstein T, Heesemann J, Roggenkamp A (2005) Nosocomial urosepsis caused by *Enterobacter kobei* with aberrant phenotype. Diagn Microbiol Infect Dis 53(2):143–147. 10.1016/j.diagmicrobio.2005.06.008 16168611

[CR8] De Maayer P, Green T, Jordan S, Smits TH, Coutinho TA (2025) Pan-genome analysis of the Enterobacter hormaechei complex highlights its genomic flexibility and pertinence as a multidrug resistant pathogen. BMC Genomics 26(1):408. 10.1186/s12864-025-11590-1 PMC12034153 40287657

[CR9] Álvarez-Marín R, Lepe JA, Gasch-Blasi O, Rodríguez-Martínez JM, Calvo-Montes J, Lara-Contreras R et al (2021) Clinical characteristics and outcome of bacteraemia caused by *Enterobacter cloacae* and *Klebsiella aerogenes*: more similarities than differences. J Glob Antimicrob Resist 25:351–358. 10.1016/j.jgar.2021.04.008 33964492

[CR10] World Health Organization (2024) WHO Bacterial Priority Pathogens List, 2024: Bacterial Pathogens of Public Health Importance to Guide Research, Development and Strategies to Prevent and Control Antimicrobial Resistance. World Health Organization, Geneva, Switzerland. https://www.who.int/publications/i/item/9789240093461 Accessed 02 November 2024

[CR11] Makinde OM, Adetunji MC, Ezeokoli OT, Odumosu BT, Ngoma L, Mwanza M, Ezekiel CN (2021) Bacterial contaminants and their antibiotic susceptibility patterns in ready-to‐eat foods vended in Ogun state, Nigeria. Lett Appl Microbiol 72(2):187–195. 10.1111/lam.13407 33030749

[CR12] Oyedele OA, Kuzamani KY, Adetunji MC, Osopale BA, Makinde OM, Onyebuenyi OE, Ogunmola OM, Mozea OC, Ayeni KI, Ezeokoli OT, Oyinloye AM (2020) Bacteriological assessment of tropical retail fresh-cut, ready-to-eat fruits in south-western Nigeria. Sci Afr 9:e00505. 10.1016/j.sciaf.2020.e00505

[CR13] Parra-Flores J, Aguirre J, Juneja V, Jackson EE, Cruz-Córdova A, Silva-Sanchez J, Forsythe S (2018) Virulence and antibiotic resistance profiles of *Cronobacter sakazakii* and *Enterobacter* spp. involved in the diarrheic hemorrhagic outbreak in Mexico. Front Microbiol 9:2206. 10.3389/fmicb.2018.02206 PMC6171480 30319560

[CR14] Wu W, Feng Y, Zong Z (2018) *Enterobacter sichuanensis* sp. nov., recovered from human urine. Int J Syst Evol MicroBiol 68(12):3922–3927. 10.1099/ijsem.0.003089 30387707

[CR15] Zhu B, Wang S, Li O, Hussain A, Hussain A, Shen J, Ibrahim M (2017) High-quality genome sequence of human pathogen *Enterobacter asburiae* type strain 1497-78T. J Global Antimicrob Resist 8:104–105. 10.1016/j.jgar.2016.12.003 28082144

[CR16] Chavda KD, Chen L, Fouts DE, Sutton G, Brinkac L, Jenkins SG, Bonomo RA, Adams MD, Kreiswirth BN (2016) Comprehensive genome analysis of carbapenemase-producing *Enterobacter* spp.: new insights into phylogeny, population structure, and resistance mechanisms. mBio 7:1–16. 10.1128/mbio.02093-16 PMC5156309 27965456

[CR17] Annavajhala MK, Gomez-Simmonds A, Uhlemann AC (2019) Multidrug-resistant *Enterobacter cloacae complex* emerging as a global, diversifying threat. Front Microbiol 10:44. 10.3389/fmicb.2019.00044 PMC6365427 30766518

[CR18] Xu T, Xue CX, Huang J, Wu J, Chen R, Zhou K (2022) Emergence of an epidemic hypervirulent clone of *Enterobacter hormaechei coproducing mcr-9* and carbapenemases. Lancet Microbe 3:e474–475. 10.1016/S2666-5247(22)00122-7 35659344

[CR19] Qiu X, Ye K, Ma Y, Zhao Q, Wang L, Yang J (2024) Genome sequence-based species classification of *Enterobacter cloacae complex*: a study among clinical isolates. Microbiol Spectr 12:e04312–e04323. 10.1128/spectrum.04312-23 PMC11237491 38687068

[CR20] Liu S, Chen L, Wang L, Zhou B, Ye D, Zheng X, Lin Y, Zeng W, Zhou T, Ye J, Liu S et al (2022) Cluster differences in antibiotic resistance, biofilm formation, mobility, and virulence of clinical *Enterobacter cloacae complex*. Front Microbiol 13:814831. 10.3389/fmicb.2022.814831 PMC9019753 35464993

[CR21] Ganbold M, Seo J, Wi YM, Kwon KT, Ko KS (2023) Species identification, antibiotic resistance, and virulence in *Enterobacter cloacae complex* clinical isolates from South Korea. Front Microbiol 14:1122691. 10.3389/fmicb.2023 PMC10076837 37032871

[CR22] Uchida A, Kim HL, Purbojati RW, Vettath VK, Gupta AB, Houghton JN, Chénard C, Wong A, Clare ME, Kushwaha KK, Panicker D (2020) Complete genome of *Enterobacter sichuanensis* strain SGAir0282 isolated from air in Singapore. Gut Pathogens 12:1–7. 10.1186/s13099-020-00350-z PMC7045367 32127921

[CR23] Adesoji AT, Marchetti VM, Cortimiglia C, Piscopiello F, Petrizzi I, Piazza A, Alabi ED, Cocconcelli PS, Migliavacca R (2025a) Antibiotic resistance and novel sequence types of klebsiella spp. in human, animal, and food sources: a one health perspective from Northern Nigeria. Curr Res Microb Sci 9:100458. 10.1016/j.crmicr.2025.100458 PMC12454290 40995512

[CR24] Adesoji AT, Alabi ED, Mattioni Marchetti V, Migliavacca R (2025b) *Serratia nevei* in Nigeria: First Report and Global Distribution. Microorganisms *13*, 2732. 10.3390/microorganisms13122732 PMC12735444 41471936

[CR26] Jain C, Rodriguez-R LM, Phillippy AM, Konstantinidis KT, Aluru S (2018) High throughput ANI analysis of 90K prokaryotic genomes reveals clear species boundaries. Nat Commun 9(1):5114. 10.1038/s41467-018-07641-9 PMC6269478 30504855

[CR27] Meier-Kolthoff JP, Auch AF, Klenk HP, Göker M (2013) Genome sequence-based species delimitation with confidence intervals and improved distance functions. BMC Bioinformatics 14:60. 10.1186/1471-2105-14-60 PMC3665452 23432962

[CR28] Jolley KA, Bray JE, Maiden MCJ (2018) Open-access bacterial population genomics: BIGSdb software, the PubMLST.org website and their applications. Wellcome Open Res 3:124. 10.12688/wellcomeopenres PMC6192448 30345391

[CR29] Kaas RS, Leekitcharoenphon P, Aarestrup FM, Lund O (2014) Solving the problem of comparing whole bacterial genomes across different sequencing platforms. PLoS ONE 9(8):e104984. 10.1371/journal.pone.0104984 PMC4128722 25110940

[CR30] Miller WR, Arias CA (2024) ESKAPE pathogens: antimicrobial resistance, epidemiology, clinical impact and therapeutics. Nat Rev Microbiol 22(10):598–616. 10.1038/s41579-024-01054-w PMC13147291 38831030

[CR31] Kaplan E, Lurie-Weinberger MN, Kastel O et al (2025) Enterobacter cloacae complex taxonomic incongruence may lead to misidentification of outbreaks and hinder infection control in real time. Eur J Clin Microbiol Infect Dis 44:3097–3101. 10.1007/s10096-025-05282-7 41023357

[CR32] Kluytmans-van den Bergh MF, Rossen JW, Bruijning-Verhagen PC, Bonten MJ, Friedrich AW, Vandenbroucke-Grauls CM, Willems RJ, Kluytmans JA (2016) Whole-Genome Multilocus Sequence Typing of Extended-Spectrum-Beta-Lactamase-Producing Enterobacteriaceae. J Clin Microbiol 54(12):2919–2927. 10.1128/JCM.01648-16 Epub 2016 Sep 14. PMID: 27629900; PMCID: PMC5121380 PMC5121380 27629900

[CR33] Oni FI, Afolayan AO, Oaikhena AO, Odih EE, Ikhimiukor OO, Ogunleye VO, Aboderin AO, Olabisi OF, Amupitan AA, Fadeyi A, Raheem RA, Olanipekun BA, Elikwu CJ, Sadare OA, Oshun PO, Oduyebo OO, Ojo F, Adeyemo AT, Mba IE, Egwuenu A, Okwor TJ, Underwood A, Argimon S, Ihekweazu C, Aanensen DM, Okeke IN (2025) Whole-genome sequencing reveals *Enterobacter hormaechei* as a key bloodstream pathogen in six tertiary care hospitals in southwestern Nigeria. Microb Genom 11(10):001508. 10.1099/mgen.0.001508 PMC12500395 41051930

[CR34] Liu W-Y, Wong C-F, Chung K-K, Jiang J-W (2013) Leung F- C. Comparative genome analysis of Enterobacter cloacae. PLoS ONE 8:e74487 10.1371/journal.pone.0074487 PMC3771936 24069314

[CR35] Maguvu TE, Bezuidenhout CC (2021) Whole genome sequencing based taxonomic classification, and comparative genomic analysis of potentially human pathogenic Enterobacter spp. isolated from chlorinated wastewater in the North West Province, South Africa. Microorganisms 9:1928. 10.3390/microorganisms9091928 PMC8466087 34576823

[CR36] Wang B, Fu J, He Y, Li K, Hu G, Hu M, Wang X, Liu W, Zhang R, Wang Y, Aimaiti B, Zhao X, Song H, Hu X, Ren H (2025) Molecular epidemiology and genomic insights into Enterobacter cloacae complex co-harbouring blaKPC and mcr: Implications for resistance gene transmission. Int J Antimicrob Agents 66(6):107602. 10.1016/j.ijantimicag.2025.107602 40885325

[CR37] Sands K, Carvalho MJ, Portal E, Thomson K, Dyer C, Akpulu C, Andrews R, Ferreira A, Gillespie D, Hender T, Hood K, Mathias J, Milton R, Nieto M, Taiyari K, Chan GJ, Bekele D, Solomon S, Basu S, Chattopadhyay P, Mukherjee S, Iregbu K, Modibbo F, Uwaezuoke S, Zahra R, Shirazi H, Muhammad A, Mazarati JB, Rucogoza A, Gaju L, Mehtar S, Bulabula ANH, Whitelaw A (2021) BARNARDS Group; Walsh TR. Characterization of antimicrobial-resistant Gram-negative bacteria that cause neonatal sepsis in seven low- and middle-income countries. Nat Microbiol 6(4):512–523. 10.1038/s41564-021-00870-7 PMC8007471 33782558

[CR38] Jesumirhewe C, Springer B, Allerberger F, Ruppitsch W (2020) Whole genome sequencing of extended-spectrum β-lactamase genes in Enterobacteriaceae isolates from Nigeria. PLoS ONE 15(4):e0231146. 10.1371/journal.pone.0231146 PMC7156064 32287306

[CR39] Yossapol M, Sugiyama M, Asai T (2017) The occurrence of CTX-M-25-producing Enterobacteriaceae in day-old broiler chicks in Japan. J Vet Med Sci 79(10):1644–1647. 10.1292/jvms.17-0294 Epub 2017 Aug 7. PMID: 28781343; PMCID: PMC5658551 PMC5658551 28781343

[CR40] Haenni M, Saras E, Ponsin C, Dahmen S, Petitjean M, Hocquet D, Madec JY (2016) High prevalence of international ESBL CTX-M-15-producing Enterobacter cloacae ST114 clone in animals. J Antimicrob Chemother 71(6):1497–1500. 10.1093/jac/dkw006 Epub 2016 Feb 4. PMID: 26850718 26850718

[CR41] Esteban-Cuesta I, Dorn-In S, Drees N, Hölzel C, Gottschalk C, Gareis M, Schwaiger K (2019) Antimicrobial resistance of Enterobacter cloacae complex isolates from the surface of muskmelons. Int J Food Microbiol 301:19–26 Epub 2019 Apr 30. PMID: 31071501 10.1016/j.ijfoodmicro.2019.04.010 31071501

[CR42] Annavajhala MK, Gomez-Simmonds A, Uhlemann AC (2019) Multidrug-Resistant *Enterobacter cloacae* Complex Emerging as a Global, Diversifying Threat. Front Microbiol 10:44. 10.3389/fmicb.2019.00044 PMC6365427 30766518

[CR43] March C, Moranta D, Regueiro V, Llobet E, Tomás A, Garmendia J, Bengoechea JA (2011) Klebsiella pneumoniae outer membrane protein A is required to prevent the activation of airway epithelial cells. J Biol Chem 286(12):9956–9967. 10.1074/jbc.M110.181008 PMC3060550 21278256

[CR44] Ievy S, Hoque MN, Islam MS, Sobur MA, Ballah FM, Rahman MS, Rahman MB, Hassan J, Khan MF, Rahman MT (2022) Genomic characteristics, virulence, and antimicrobial resistance in avian pathogenic Escherichia coli MTR_BAU02 strain isolated from layer farm in Bangladesh. J Global Antimicrob Resist 30:155–162. 10.1016/j.jgar.2022.06.001 35671989

[CR45] Rahi P, Mühle E, Scandola C, Touak G, Clermont D (2024) Genome sequence-based identification of Enterobacter strains and description of Enterobacter pasteurii sp. nov. Microbiol Spectr 12(1):e03150–e03123. 10.1128/spectrum.03150-23 PMC10783019 38099614

[CR46] Folgori L, Di Carlo D, Comandatore F, Piazza A, Witney AA, Bresesti I, Hsia Y, Laing K, Monahan I, Bielicki J, Alvaro A (2021) Antibiotic susceptibility, virulome, and clinical outcomes in european infants with bloodstream infections caused by enterobacterales. Antibiotics 10(6):706. 10.3390/antibiotics10060706 PMC8230887 34208220

[CR47] Bolourchi N, Giske CG, Nematzadeh S, Mirzaie A, Abhari SS, Solgi H, Badmasti F (2022) Comparative resistome and virulome analysis of clinical NDM-1–producing carbapenem-resistant Enterobacter cloacae complex. J Global Antimicrob Resist 28:254–263. 10.1016/j.jgar.2022.01.021 35121164

[CR48] Lumbreras-Iglesias P, de Toro M, Vázquez X, García-Carús E, Rodicio MR, Fernández J (2023) High-risk international clones ST66, ST171 and ST78 of Enterobacter cloacae complex causing blood stream infections in Spain and carrying bla_OXA–48_ with or without mcr-9. J Infect Public Health 16(2):272–279. 10.1016/j.jiph.2022.12.015 36621205

[CR49] Ballash GA, Mathys DA, Feicht SM, Mollenkopf DF, Albers AL, Adams RJ, Wittum TE (2023) Antimicrobial-Resistant *Enterobacterales* Recovered from the Environment of Two Zoological Institutions Include Enterobacter cloacae Complex ST171 Producing KPC-4 Carbapenemase. Appl Environ Microbiol 89(5):e0025723. 10.1128/aem.00257-23 PMC10231243 37067417

[CR50] Ghazawi A, Anes F, Mouftah S, Elbediwi M, Baig A, Alketbi M, Almazrouei F, Alhashmi M, Alzarooni N, Manzoor A, Habib I, Strepis N, Nabi A, Khan M (2024) Genomic Study of High-Risk Clones of *Enterobacter hormaechei* Collected from Tertiary Hospitals in the United Arab Emirates. Antibiot (Basel) 13(7):592. 10.3390/antibiotics13070592 PMC11274081 39061274

[CR51] Kim JJ, Ryu S, Lee H (2018) Foodborne illness outbreaks in Gyeonggi province, Korea, following seafood consumption potentially caused by Kudoa septempunctata between 2015 and 2016. Osong public health Res Perspect 9(2):66. 10.24171/j.phrp.2018.9.2.05 PMC5935147 29740530

[CR52] Garinet S, Fihman V, Jacquier H, Corvec S, Le Monnier A, Guillard T, Cattoir V, Zahar JR, Woerther PL, Carbonnelle E, Wargnier A (2018) Elective distribution of resistance to beta-lactams among Enterobacter cloacae genetic clusters. J Infect 77(3):178–182. 10.1016/j.jinf.2018.05.005 29807092

[CR53] Pot M, Reynaud Y, Couvin D, Ducat C, Ferdinand S, Gravey F, Gruel G, Guérin F, Malpote E, Breurec S, Talarmin A (2021) Wide distribution and specific resistance pattern to third-generation cephalosporins of Enterobacter cloacae complex members in humans and in the environment in Guadeloupe (French West Indies). Front Microbiol 12:628058. 10.3389/fmicb.2021.628058 PMC8268024 34248862

[CR54] Chang CY, Huang PH, Lu PL (2022) The Resistance Mechanisms and Clinical Impact of Resistance to the Third Generation Cephalosporins in Species of Enterobacter cloacae Complex in Taiwan. Antibiotics 11(9):1153. 10.3390/antibiotics11091153 PMC9494969 36139933

[CR55] Haenni M, Saras E, Ponsin C, Dahmen S, Petitjean M, Hocquet D, Madec JY (2016) High prevalence of international ESBL CTX-M-15-producing Enterobacter cloacae ST114 clone in animals. J Antimicrob Chemother 71(6):1497–1500. 10.1093/jac/dkw006 26850718

[CR56] Focker M, van der Fels-Klerx HJ (2020) Economics applied to food safety. Curr Opin Food Sci 36:1823. 10.1016/j.cofs.2020.10.018

[CR57] Donà V, Nordmann P, Kittl S, Schuller S, Bouvier M, Poirel L, Endimiani A, Perreten V (2023) Emergence of OXA-48-producing Enterobacter hormaechei in a Swiss companion animal clinic and their genetic relationship to clinical human isolates. J Antimicrob Chemother 78(12):2950–2960. 10.1093/jac/dkad337 37923369

[CR58] Sadek M, Nariya H, Shimamoto T, Kayama S, Yu L, Hisatsune J, Sugai M, Nordmann P, Poirel L, Shimamoto T (2020) First genomic characterization of *bla*_VIM–1_ and *mcr-9-*coharbouring *Enterobacte rhormaechei* Isolated from food of animal origin. Pathogens (Basel, Switzerland) 9:687. 10.3390/pathogens9090687 PMC7558541 32842587

[CR59] Macesic N, Blakeway LV, Stewart JD, Hawkey J, Wyres KL, Judd LM, Wick RR, Jenney AW, Holt KE, Peleg AY (2021) Silent spread of mobile colistin resistance gene mcr-9.1 on IncHI2 ‘superplasmids’ in clinical carbapenem-resistant Enterobacterales. Clin Microbiol Infect 27(12):1856–e7. 10.1016/j.cmi.2021.04.020 33915285

[CR60] Algarni S, Gudeta DD, Han J, Nayak R, Foley SL (2024) Genotypic analyses of IncHI2 plasmids from enteric bacteria. Sci Rep 14(1):9802. 10.1038/s41598-024-59870-2 PMC11058233 38684834

